# Isolation of a New Mexican Strain of *Bacillus subtilis* with Antifungal and Antibacterial Activities

**DOI:** 10.1100/2012/384978

**Published:** 2012-04-19

**Authors:** M. G. L. Basurto-Cadena, M. Vázquez-Arista, J. García-Jiménez, R. Salcedo-Hernández, D. K. Bideshi, J. E. Barboza-Corona

**Affiliations:** ^1^División de Ciencias de la Vida, Departamento de Alimentos, Universidad de Guanajuato Campus Irapuato-Salamanca, 36500, Irapuato, GTO, Mexico; ^2^Departamento de Ecosistemas Agroforestales, Universidad Politécnica de Valencia, Camino de Vera, S/N, 46022 Valencia, Spain; ^3^Department of Natural and Mathematical Sciences, California Baptist University, 8432 Magnolia Avenue, Riverside, CA 92504, USA; ^4^Department of Entomology, University of California, Riverside, Riverside, CA 92521, USA

## Abstract

Although several strains of *B. subtilis* with antifungal activity have been isolated worldwide, to date there are no published reports regarding the isolation of a native *B. subtilis* strain from strawberry plants in Mexico. A native bacterium (*Bacillus subtilis* 21) demonstrated *in vitro* antagonistic activity against different plant pathogenic fungi. Under greenhouse conditions, it was shown that plants infected with *Rhizoctonia solani* and *Fusarium verticillioides* and treated with *B. subtilis* 21 produced augment in the number of leaves per plant and an increment in the length of healthy leaves in comparison with untreated plants. In addition, *B. subtilis* 21 showed activity against pathogenic bacteria. Secreted proteins by *B. subtilis* 21 were studied, detecting the presence of proteases and bacteriocin-like inhibitor substances that could be implicated in its antagonistic activity. Chitinases and zwittermicin production could not be detected. Then, *B. subtilis* 21 could potentially be used to control phytopathogenic fungi that infect strawberry plants.

## 1. Introduction

Data published by the Food and Agriculture Organization [1] indicated that Mexico is among the tenth largest producers of strawberries worldwide. In Mexico, the State of Guanajuato is one of the major producers of this crop, but during the past few years different factors, including the “secadera” disease, have led to an *∼*50% decline in productivity [2]. The etiologic agents of “secadera” are fungi, primarily *Rhizoctonia *sp., *Fusarium* sp., *Verticillium* sp., and *Phytophthora* sp. [3]. 

In Mexico, chemical pesticides are used to control agricultural pests, but their long-term effects on animals and the environment have stimulated much concern [4]. To circumvent known and proposed detrimental effects related to widespread use of these pesticides, much interest is focused in identifying and developing biological agents to control insects and phytopathogenic microbes [5]. In particular, the use of selected nonpathogenic microorganisms which are ubiquitous in the soil and has potential applied use if they naturally produce antagonistic metabolites that kill bacterial or fungal pathogens or significantly inhibit their proliferation. For example, it has been demonstrated that an antibiotic isolated from *Bacillus cereus* inhibited mycelial growth of *Fusarium roseum*, and chitinases synthesized by *Serratia marcescens*, *Streptomyces* spp. *Bacillus circulans,* and *Trichoderma harzianum* are involved in the degradation of the host fungus cellular walls [6]. 

Volatile antimicrobials compounds produced by the endophytic fungus *Muscodor albus* are also known to kill a broad range of fungi and bacteria that are pathogenic to plants and humans [7]. Indeed, both diffusible and volatile compounds, bacteriocins, siderophores, chitinases, cellulases, amylases, lipopeptide antibiotics (e.g., fengycin, surfactin, iturin), and nonlipopeptide antibiotics (e.g., sublacin, subtilin, subtilosin) are examples of other molecules produced by microbes that interfere with the establishment and survival of microbial ecological communities [8, 9]. 

Interestingly, wild type and recombinant strains of *Bacillus subtilis* are known to not only promote plant growth but also synthesize different metabolites with antibacterial and antifungal activities [9]. As such, natural strains of probiotic *B. subtilis* could be beneficial as biocontrol agent in the strawberry industry. However, though many different environmental isolates and strains of *B. subtilis* have been described, to date there is no report regarding the identification of native *B. subtilis* isolates from strawberries in Mexico that elicit significant antagonistic activity against fungi that cause the “secadera” disease [10]. 

In this study, we isolated and cultured a new antifungal isolate of *B. subtilis* (isolate 21) from strawberries grown in Irapuato Guanajuato, Mexico, that showed antagonistic effect *in vitro *and under greenhouse conditions to pathogenic fungi.

## 2. Material and Methods

### 2.1. Microbial Strains

*Bacillus thuringiensis* subsp. *morrisoni* (LBIT 269) was obtained from a native bacterial stock collection held at CINVESTAV, Campus Guanajuato, Mexico. This strain synthesizes the bacteriocin called morricin 269 [11]. *Bacillus cereus* 183 was obtained from a collection of *Bacillus* strains maintained in the International Entomopathogenic *Bacillus* Centre, Institute Pasteur, Paris, France, and was employed as the indicator bacterium for determining, by a fluorogenic method, the time of the highest bacteriocin production by *B. subtilis *21 [12]. Antibacterial activity of morricin 269 and bacteriocin-like inhibitor substances (BLISs) produced by *B. subtilis *21 (Bs21-BLIS) were evaluated against Gram-positive bacteria, *Staphylococcus xylosus* ATCC 700404, *Staphylococcus aureus* ATCC 25923, *Listeria innocua*, *Bacillus cereus *183, *Streptococcus pyogenes*, and *Streptococcus pneumonia*, and Gram-negative bacteria, *Pseudomona aeruginosa* ATCC 27853, *Enterobacter cloacae* ATCC 13047, *Proteus vulgaris* ATCC 13315, *Escherichia coli* (Quanti-Cult), *Salmonella* sp., and *Shigella flexneri*. *Salmonella* sp. and *L. innocua* were obtained from the Laboratory of Public Health of the State of Hidalgo (LPHSH), Mexico. Bacteria not obtained from an ATCC collection or LPHSH were acquired from the Clinical Microbiological Laboratory and Sanitary of the Laguna, Coahuila, Mexico [13].

### 2.2. Isolation and Bacterial Identification

Soil samples collected from the rhizosphere of healthy strawberry plants grown in different localities in Irapuato Guanajuato, Mexico, were homogenized to obtain uniform particle size. Crown and roots of the same plants were cut into 1 cm pieces, washed with 1.5% (v/v) sodium hypochlorite, and rinsed several times with distilled water. Homogenized soil was inoculated onto PDA (potato dextrose agar, Bioxon) using a small cylindrical sponge. Crown and roots pieces were also grown on the same media and incubated at room temperature (25–28°C). Axenic bacterial cultures were maintained on PDA slants. Bacterial identification was performed by general and specific biochemical tests to determine the genus and the species [14].

### 2.3. Isolation and Pathogenic Fungi Identification

Crown and roots of strawberry plants with the “secadera” disease were washed with distilled water to eliminate soil and cut into ~1 cm pieces. Subsequently, pieces were disinfected with 1.5% (v/v) sodium hypochlorite, rinsed twice with distilled water, and placed in plates with PDA supplemented with 10% (w/v) lactic acid. Plates were incubated at 22°C or 28°C for *Rhizoctonia* species and other fungi, respectively. Fungal identification was performed as previously described [15, 16].

### 2.4. *In Vitro* Antifungal Activity

Agar disks from axenic fungus culture were inoculated on the middle of Petri dishes and cultivated at 28°C. When mycelium reached about 2 cm of radial grow, *B. subtilis* 21 was inoculated at approximately 4.5 cm from the fungal margin and incubated at the same temperature to determine the formation of an inhibition zone. *Bacillus subtilis* 1.2.2 was used for comparison [17].

### 2.5. Antifungal Activity of *Bacillus subtilis* 21 in Greenhouse Environment

Antagonism assays with both *R. solani* and *F. verticillioides* were carried out for a period of 90 days under greenhouse conditions [18]. To evaluate antagonism activity, the number of strawberry leaves without “secadera” disease symptoms and their length in centimeters were recorded. Treatments were untreated plants; strawberry plants infected with *R. solani* or *F. verticillioides* and treated separately with *B. subtilis* 21, *B. subtilis*1.2.2, and the fungicide Busan 30WB (2-tiocianometiltiobenzotiazol) (TCMTB). Roots plants were submerged for 1 min in suspensions of *B. subtilis* 21 or *B. subtilis* 1.2.2 containing ~1 × 10^7^ cells/mL or in 50 ppm of the fungicide TCMTB before planted. All assays were carried out in triplicate (three pods with three strawberry plants/pot) and a mix of vermiculate, pro-moss, and organic soil (1 : 1 : 2) as substrate was used [10]. Analysis of variance (ANOVA) was performed using the statistical model for a completely randomized block design. Treatment means were compared using the Tukey's test at significance level of 0.05%.

### 2.6. Determination of Chitinolytic Activity

Bacteria were cultivated overnight (28°C, 200 rpm) in LB (Luria-Bertani) medium without antibiotic to achieve an absorbance of ~1.7 at 600 nm. Then 500 *μ*L of cultures containing ~1 × 10^9^ cells/mL were transferred to 100 mL of liquid Castañeda medium [0.06% (w/v) ammonium citrate, 0.02% (w/v) NaCl, 0.04% (w/v) KH_2_PO_4_, 0.01% (w/v) MgSO_4_·7H_2_O, 0.04% (w/v) Na_2_CO_3_, pH 7] supplemented with either 0.5% (w/v) colloidal chitin or 0.3% (w/v) of dry and sterilized *Rhizoctonia solani* mycelium. Aliquots were taken at different intervals (24 h, 48 h, and 72 h) and centrifuged, and the supernatants containing secreted proteins were assayed for chitinase activity by triplicates using the fluorogenic substrates 4-methylumbelliferyl-*β*-D-*N*, *N*′, *N*′′*-*triacetylchitotriose [4-MU-(GlcNAc)_3_], 4-methylumbelliferyl-*β*-D-*N*, *N*′*-*diacetylchitobioside [4-MU-(GlcNAc)_2_], and 4-methylumbelliferyl-*N*-acetyl-*β*-D-glucosaminide [4-MU-GlcNAc] (Sigma) to detect endochitinases, exochitinases, and *N*-acetylglucosaminidases, respectively. Reaction mixtures were prepared as described previously, diluted appropriately, and incubated at 37°C for at least 15 min. Chitinase activity was measured spectrophotofluorometrically (excitation 340 nm, emission 415 nm). One unit of enzyme activity was defined as the amount of enzyme required to release 1 *μ*mol of 4-methylumbelliferone (MU) in 1 h [19]. Additionally, 72 h cultures were centrifuged, and ammonium sulfate was added to supernatant at 80% saturation to concentrated extracellular proteins with constant stirring overnight. Concentrated proteins were resuspended in 2 mL of 100 mM phosphate buffer and dialyzed overnight against the same buffer using membranes of 10 kDa cut-off (Spectrum Laboratories Company). Chitinase activity was determined as described above using fluorogenic substrates [19, 20].

### 2.7. Proteolytic Activity

Bacteria were cultivated in 100 mL of liquid Castañeda medium supplemented with 1% (w/v) casein under the same conditions described above for chitinolytic activity determination. One hundred microliters of cultures were collected at 24, 48, and 72 h, centrifuged and supernatants mixed with 0.4 mL of double-distilled water, 1 mL of 1% (w/v) casein in 200 mM glycine-NaOH buffer (pH 9), and incubated at 37°C for 30 min. Then, reactions were stopped by adding 3.5 mL of 4% (w/v) trichloroacetic acid, centrifuged, and the absorbance was measured at 280 nm. A standard curve of tyrosine (0–300 *μ*M/mL) was carried out to determine the protease units (PU). One PU was defined as the amount of enzyme required to release 1 *μ*g of tyrosine/min [21].

### 2.8. Antibacterial Activity

To study the kinetics of bacteriocin-like inhibitor substances produced by *B. subtilis *21 (Bs21-BLIS), bacterium was cultured in TSB (tryptic soy broth) at 28°C and samples were assayed in duplicate at different times over a 72 h period. One of the samples was used for monitoring cell growth spectrophotometrically at 600 nm, and the other was employed for bacteriocin detection using a rapid fluorogenic method [12], using *B. cereus *183 as indicator bacterium. After determining the time that yielded the highest level of bacteriocin production, the bacterium was cultivated in fresh TSB and culture supernatant was concentrated with ammonium sulfate as described previously [12, 13]. Precipitated proteins were pelleted by centrifugation at 16.000× g for 30 min at 4°C, resuspended in 100 mM phosphate buffer (pH 7.0), and dialyzed overnight against the same buffer using a minidialysis kit with a 1 kDa cut-off (Amersham Biosciences). Antibacterial activity of concentrated proteins was tested using the modified well-diffusion method [12]. Briefly, twenty-five ml of TSB with soft agar 0.7% (w/v) was mixed with 50 *μ*L (~1 × 10^9^ cells/mL) of indicator bacteria and plated. Wells, 7 mm in diameter, were dug into the agar and stored for 2 h at 37°C to dry the humidity. Then 100 *μ*L of the concentrated proteins were added to each well and incubated for 12 h at 4°C to allow the diffusion of the samples followed by an additional incubation at 28°C for 1 day before diameters of zones of inhibition were measured. The minimum detectable zone measured for analytic purposes was 1 mm beyond the well diameter. Each point of activity was repeated in triplicate and the average was recorded. For our purposes, we define one unit (U) of bacteriocin activity as equal to 1 mm^2^ of the zone of inhibition of growth of the indicator bacterium [12, 13].

### 2.9. Detection of Zwittermicin A Gene

DNA preparations were obtained [22]. Oligonucleotides A0678 (5′-ATGTGCACTTGTATGGGCAG-3′) and A0677 (5′-TAAAGCTCGTCCCTCTTCAG-3′) were used as forward and reverse primers [23, 24]. Gene amplification was performed with the PCR Reagent System (Invitrogen) in a thermocycler (iCycler Bio-Rad) for 30 cycles as follows: 94°C for 15 s, 55°C for 45 s, 72°C for 2 min, followed by a 4 min termination cycle at 72°C*. B. thuringiensis *subsp.* morrisoni *(LBIT 269) and *B. cereus* 183 were used as positive controls for zwittermycin gene amplification [11].

## 3. Results

### 3.1. Pathogenic Fungi Isolation and Identification

Two hundred and twenty-six morphologically distinct fungal colonies were isolated from strawberry with the “secadera” disease. From those isolates, 63 were selected based on their morphology and microscopic characteristics and placed into three groups. Group 1 was composed of 33 strains (52.4%) of *Fusarium verticillioides* (Sacc.) Nuremberg and had purple cottony aerial mycelia and sickle-shaped thin-walled macro conidia. In the second group, 21 isolates (33.3%) of *Rhizoctonia solani* Kühn were included as they showed aerial mycelia in radial layers and segmented hyphae with “T” joints. Finally, 9 isolates (14.2%) that could not be identified comprised Group 3 [10].

### 3.2. Bacteria Isolation, Identification, and Antagonistic Activity

Sixteen hundred bacterial colonies were cultured from healthy strawberry plants and used in antagonism assays against *R. solani* and *F. verticillioides*. From these, 8 bacterial isolates (*B. cereus, B. licheniformis, B. subtilis, Chromobacterium lividum, Flavobacterium *sp*., Janttinobacterium *sp*., P. aeruginosa, *and* S. marcescens*) (Table 1) with antagonistic activity to both fungi were selected for further studies. Antifungal activity was compared with that obtained with *B. subtilis *1.2.2 [10]. From these, an isolate of *B. subtilis*, hereafter *B. subtilis *21, showed the highest antifungal activity (Table 1). Additionally, *B. subtilis *21 showed inhibitory activity to different cash crop pathogenic fungi isolated from Spain (*Verticillium* spp. *Rosellinia necatrix, Armillaria mellea,* and *Alternaria alternata* pv. citri) (Table 2). 

### 3.3. Greenhouse Assays

As indicated above, healthy strawberry plants were infected with *R. solani *and* F. verticillioides* and then subjected to the treatment with *B*. *subtilis* 21 and *B*. *subtilis* 1.1.2 and the TCMTB fungicide. The results of this study were analyzed after 90 days. When infected plants were exposed to *B. subtilis*, they developed more leaves than those exposed to the TCMTB fungicide and also they were larger (see below). It has been previously reported that some strains of *B. subtilis *secrete compounds that could act as growth promoters [9], suggesting that *B. subtilis *21 might liberate growth regulators. When strawberry plants were infected with *R. solani* and then treated with *B. subtilis* 21, 1.2.2 and also with the TCMTB fungicide, they did not show any apparent difference before 45 days of the assay, but after that time there were statistically significant differences, at 0.05%, in the number of healthy leaves until the end of the experiment. When infected plants with *R. solani* were treated with *B. subtilis* 21, scores of 5 to 6 strawberry leaves per plant did not have disease symptoms, whereas only 1 to 2 healthy leaves were observed if infected plants were treated with *B. subtilis* 1.2.2 or the TCMTB fungicide. The length of strawberry leaves was also measured after 90 days of the biological and chemical control application. The leaves of infected plants treated with *B. subtilis *21 had an average length of 4.5 cm, whereas with *B. subtilis* 1.2.2 and the TCMTB fungicide values were approximately 1.5 cm. Similarly, when strawberry plants were infected with *F. verticillioides* and exposed to bacteria and the fungicide, we found visible differences in the number of infected leaves. If plants were assayed with *B. subtilis* 21 and *B. subtilis* 1.2.2, scores of 7 to 9 strawberry leaves per plants were without disease symptoms, compared with 2 to 3 for the TCMTB fungicide treatment. Additionally, length of strawberry leaves was between 4 and 6 cm if they were subject to *B. subtilis* 21 and *B. subtilis* 1.2.2, but if plants were treated with TCMTB fungicide, their length was of 1 and 2 cm [10].

### 3.4. Chitinolytic and Proteolytic Activity Determination

In order to test whether *B. subtilis *21 was able to synthesize chitinase, the bacterium was grown in two media containing salts and varying the carbon and nitrogen sources. The media were supplemented with either colloidal chitin or dry mycelium of *Rhizoctonia solani. *No chitinase production was detected in both media. In addition, *B. subtilis *21 synthesized proteases during growth but the maximum production (~450 UP/mL/min) was detected during the middle of the logarithmic phase ~20 h (data not shown).

### 3.5. Bactericidal Activity

**Figure 1:**
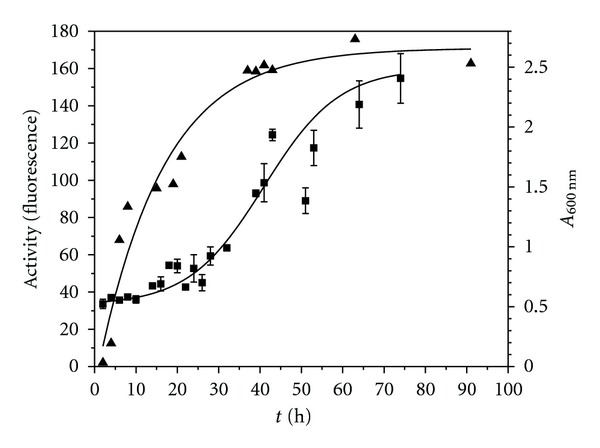
Correlation between growth and the appearance of Bs21-BLIS in the culture medium. Bacterium was grown in tryptic soy broth, and duplicate samples were collected at different times. One sample was used for measuring the optical density at 600 nm (▲) and the other for evaluating the Bs21*-*BLIS activity (■) against *Bacillus cereus *employing a fluorogenic rapid method.

Antibacterial activity was evaluated using a fluorogenic rapid method [12], employing *B. cereus *183 as indicator bacterium (Figure 1). In this assay, bacteriocin activity was observed in samples collected at the middle of the logarithmic phase and achieved the highest levels at the start of the stationary period (Figure 1). Subsequently, Bs21-BLIS was concentrated at the time corresponding to the highest level of bacteriocin production. Samples were evaluated against different Gram-positive and Gram-negative bacteria known to be etiologic agents of human diseases with the well-diffusion method and activities were compared to that of morricin 269 (Table 3). This bacteriocin showed a wide spectrum of antibacterial inhibitory effect whereas Bs21-BLIS had a narrow inhibitory effect obtaining the highest activity against *B. cereus *183 (276 U) and *Staphylococcus aureus *(276 U), followed by *Streptococcus pyogenes *(25 U) and *Enterobacter cloacae* (25 U).

### 3.6. Zwittermicin A Gene Identification

**Figure 2:**
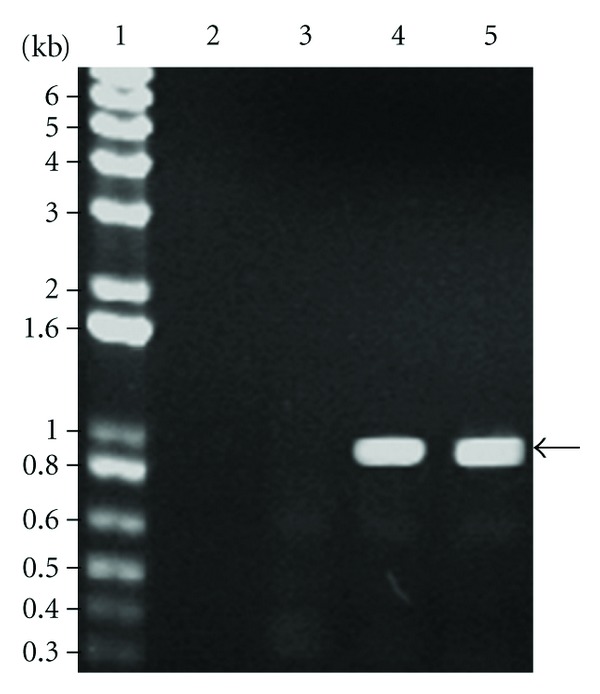
Amplification of zwittermycin A gene by the polymerase chain reaction. Lane 1, 1 kbp plus DNA ladder (Invitrogen); lane 2, control without DNA; lane 3, *B. subtilis *21; lane 4, *B. thuringiensis *subsp. *morrisoni *(LBIT 269); lane 5, *B. cereus *183. Arrow indicates the position of an amplicon of ~0.9 Kb corresponding to the zwittermycin A gene.

Amplicons of ~0.9 kb corresponding to the expected size for a homologue zwittermicin A gene were obtained with *B. thuringiensis *subsp. *morrisoni *(LBIT 269) and *B. cereus *183 [11] (Figure 2); however a corresponding amplicon was not observed for *B. subtilis *21.

## 4. Discussion

One of the most important factors that have contributed to the decline in strawberry production in Guanajuato, Mexico, over the past few years is the infection of plants caused by diverse phytopathogenic fungi. Because of the complications posed by chemical pesticide use, new natural biological control approaches are being considered to control agricultural pests. With regard to phytopathogenic fungi, although several strains of *B. subtilis *with antifungal activity have been isolated worldwide, to date there are no published reports regarding the isolation of a native *B. subtilis *strain with activity to fungi associated with disease of strawberry plants. At the start of this study, we decided that comparative screening of healthy and unhealthy strawberry plants could aid in identifying native bacteria capable of inhibiting growth of fungi responsible for the “secadera” disease. From 1600 isolates, we were able to identify 8 bacteria with *in vitro* activity against native *F. verticillioides *and *R. solani *isolates. Of these bacteria, *B. subtilis *21 showed the highest activity, more than *B. subtilis *1.2.2, a bacterium previously employed as positive control in antagonism assays. The antifungal activity of *B. subtilis *21 observed against species that cause different cash crops disease in Spain (*Verticillium* spp, *Rosellinia necatrix, Armillaria mellea,* and *Alternaria alternata* pv *citri*) could be a significant finding as it suggests that the bacterial isolate could have widespread biocontrol utility. Importantly, after demonstrating *in vitro* activity, we showed that *B. subtilis* 21 was also able to inhibit the growth of *R. solani* and *F. verticillioides* under greenhouse conditions. In general, infected plants inoculated with *B. subtilis *21 statistically showed less pathologic effects when compared with infected plants treated with *B. subtilis *1.2.2 and the fungicide TCMTB; results that imply that the antifungal bacterium could have applied use.

Antimycotic activity is known to be induced by chitinases produced by a variety of microorganisms including *Enterobacter agglomerans *[25] and *Trichoderma harzianum *[6]. It appears that the antifungal activity of *B*. *subtilis *21 is not due to constitutive or induced chitinase as such activity was not detected in our assays using preparations of the bacterium obtained from growth media supplemented with chitin or *Rhizoctonia solani* mycelia or without these inducers. Additionally, the role of subtilisin-like proteases with activity against *Alternaria alternata, Fusarium oxysporum, Rhizoctonia solani, Sclerotinia sclerotiorum,* and *Cytospora chrysosperma* has been demonstrated with *Trichoderma harzianum *T88 [26]. Our results showed that antifungal protease production occurred during all phases of *B. subtilis *21 growth which suggests that those organic molecules could play an important role in its antifungal activity. 

Other compounds that confer antimycotic activity include zwittermicin A, an aminopolyol antibiotic encoded by the zwittermicin A gene in *Bacillus* species such as *B*. *cereus *UW85 [23, 24]. Our inability to produce an amplicon corresponding to the *B. cereus* UW85 zwittermicin A gene in *B. subtilis* 21 suggests that a closely related homolog is absent in this isolate. However, it is possible that a more diverged gene not amplifiable with the primers used in this study could be present in the isolate. Therefore we are unable to conclude that the antifungal activity of *B. subtilis* 21 is not due to a zwittermicin-A-like antibiotic. 

Studies on the kinetics of bacteriocins production by *B. subtilis *21 were very similar to those observed for *B. thuringiensis *subsp.* kenyae *(LBIT 404), *B. thuringiensis *subsp. *entomocidus *(LBIT 420), and *B. thuringiensis *subsp. *tolworthi *(LBIT 524), where bactericidal activity was present in samples collected at middle of the logarithmic phase of growth and achieved the highest levels at the start of the stationary period Barboza-Corona et al. [11]. The Bs21-BLIS activity against *Staphylococcus aureus*, *Streptococcus pyogenes, *and *Enterobacter cloacae, *potent agents of a number of community acquired and nosocomial diseases, including emesis, diarrhea, sore throat and scarlet fever, and urinary tract infections [27], suggests that these substances could be useful as a preservative in stored consumable products. 

In conclusion, we cultured an isolate *B. subtilis* 21 that showed inhibitory activity *in vitro* and under greenhouse conditions against several fungi of economic importance including *Verticillium* sp. and agent of “secadera disease” and also showed that it produces extracellular compounds, such as proteases and bacteriocin-like inhibitor which could be implicated in the antagonistic activity against fungi and food-borne pathogenic bacteria.

Future efforts will focus on two important items: (a) identifying specific substance produced by *B*. *subtilis* 21, including volatile substances, that could play an important role in its antifungal activity and could be important in controlling fungal agents of “secadera disease” and (b) performing antagonistic assays against fungi under field conditions.

## Figures and Tables

**Table 1:** Antifungal activity of bacteria isolated from strawberry plants^a^.

Bacteria	Strain	Fungi	
		*F. verticillioides*	*R. solani*
*Bacillus cereus*	6	+	+
*B. licheniformis*	99	+	+
*B. subtilis*	21	+ ++	+ ++
*Chromobacterium lividum*	17	+	+
*Flavobacterium (*C group)	65	+	+ +
*Jantinobacterium *sp	35	+	+
*Pseudomonas aeruginosa*	4	+	+
*Serratia marcescens*	77	+	+
*B. subtilis*	1.2.2	+ +	+ +

^a^The intensity of the antagonistic activity was recorded on basis of the size of growth inhibition from the place where bacterium was inoculated to the edge of the spreading fungal mycelium as follows: strong antagonism (+++), middle antagonism (++), and light antagonism (+) if the fungal growth was, respectively, ≤3.5 cm, 3.5 to 4.5 cm, or >4.5 cm.

**Table 2:** Inhibitory activities of *B. subtilis* 21 against pathogenic fungi isolated from Spain cash crops.

Fungi	Strains	Cash crops	Antagonistic activity^a^
*Verticillium* sp.	V1-S1	Watermelon	+ ++
*Verticillium* sp.	V1-S2	Watermelon	+ +
*Verticillium *sp.	V2-S3	Watermelon	+ ++
*Verticillium* sp.	V2-S4	Watermelon	+ ++
*Verticillium *sp.	V4-C1	Cucumber	+ ++
*Verticillium *sp.	V4-T1	Tomato	+ +
*Verticillium* sp.	V5-P1	Pumpkin	+ ++
*Rosellinia necatrix*	P4-A1	Nispero	+ ++
*Rosellinia necatrix*	P4-A2	Nispero	+ ++
*Armillaria mellea*	A3-M1	Nispero	+ ++
*Armillaria mellea*	A3-M2	Nispero	+ +
*Armillaria mellea*	A3-M3	Nispero	+ +
*Armillaria mellea*	A5-M4	Nispero	+ ++
*Armillaria mellea*	A5-M5	Nispero	+ ++
*Armillaria mellea*	A5-M6	Nispero	+ ++
*Armillaria mellea*	A6-M7	Nispero	+ ++
*Armillaria mellea*	A6-M8	Nispero	+ ++
*Armillaria mellea*	A7-M9	Nispero	+ ++
*Alternaria alternata* pv citri	—	Mandarin	+ ++

^a^Strong antagonism (+++), middle antagonism (++), and light antagonism (+) if the fungal growth was, respectively, ≤3.5 cm, 3.5 to 4.5 cm, or >4.5 cm.

**Table 3:** Antibacterial activity (*U* ^a^) of partial purified bacteriocins from *B. subtilis *21 (Bs21-BLIS).

Indicator bacteria	Bs21-BLIS	Morricin 269^b^
Gram-positive		
*Bacillus cereus 183*	276	402
*Listeria innocua*	0	610
*Staphylococcus* *aureus *	276	1040
*Staphylococcus* *xylosus *	0	610
*Streptococcus* *pneumonia *	0	0
*Streptococcus* *pyogenes *	25	104
Gram-negative		
*Enterobacter cloacae*	25	441
*Escherichia coli*	0	204
*Proteus vulgaris*	0	0
*Pseudomona aeruginosa*	0	610
*Salmonella *sp.	0	264
*Shigella flexneri*	0	63

^a^One unit is defined as 1 mm^2^ of the zone of inhibition as determined by the well-diffusion method (see text). Data are the average of triplicate tests. A value of “0” indicates no inhibition.

^b^Production of morricin 269 by LBIT 269 was performed as described [13].
